# Poly(ethylene oxide) Is Positively Charged in Aqueous Solutions

**DOI:** 10.3390/gels8040213

**Published:** 2022-03-31

**Authors:** Chao Zhou, Chunda Ji, Yuchen Nie, Jingfa Yang, Jiang Zhao

**Affiliations:** 1 Beijing National Research Center for Molecular Sciences, Institute of Chemistry, Chinese Academy of Sciences, Beijing 100190, China; zhouchao@iccas.ac.cn (C.Z.); jichunda@iccas.ac.cn (C.J.); nieyuchen19@mails.ucas.ac.cn (Y.N.); yangjf@iccas.ac.cn (J.Y.); 2 School of Chemical Sciences, University of Chinese Academy of Sciences, Beijing 100049, China

**Keywords:** polyethylene oxide, charged, single molecule fluorescence, cations

## Abstract

There have been controversies about the binding of cations to poly(ethylene oxide) (PEO) chains in aqueous solutions. In the current study, single molecular evidence of charging PEO chains by cation binding in aqueous solutions is provided. From the adoption of the photon-counting histogram method, it is discovered that the local pH value at the vicinity of the PEO chain is higher than the bulk solution, showing that the PEO chain is positively charged. Such a situation exists with and without the presence of salt (NaCl) in the solution, presumably due to the binding of cations, such as hydronium and sodium ions. Single molecular electrophoresis experiments using fluorescence correlation spectroscopy demonstrate that the PEO chains are weakly charged with a charging extent of ~5%. In comparison to the salt-free condition, the addition of external salt (NaCl) at moderate concentrations further charges the chain. The charging causes the PEO chains to expand and a further increase in the salt concentration causes the chain to shrink, exhibiting a polyelectrolyte-like behavior, demonstrated by the hydrodynamic radii of a single PEO chain. The effect of ion identity is discovered with alkali cations, with the order of the charging capacity of Li^+^ < Na^+^ < Cs^+^ < K^+^.

## 1. Introduction

The understanding of ion binding to macromolecules and the associated consequences is of fundamental relevance to technological and biological applications, from battery performance, biomedical application, to biological processes [1,2,3]. Being an important polymer for bio-medical applications, such as hydrogels for drug delivery, bio-conjugation, surface modification, and tissue engineering, poly(ethylene oxide) (PEO) and its capacity of interacting with electrolytes has been attracting the attention of researchers for decades [4,5,6,7,8,9,10,11,12]. It has been discovered that, in solvents with a moderate dielectric constant and moderate hydrogen-bonding capacity, such as methanol, the addition of monovalent ions can cause the PEO chain to be charged at low ionic strengths due to the PEO–cation association, causing the chain to expand as a consequence. Such an effect weakens under the condition of high ionic strength due to the screening of electrostatic interactions [13,14,15]. Ion binding to the PEO chains becomes too weak to exert a charging effect in solvents of a high dielectric constant or pronounced hydrogen-bonding capacity, such as water, and no polyelectrolyte-like behavior is exhibited, as investigated in scattering experiments [14,15]. Yet, there has been a report on the evidence of cation binding to PEO chains in aqueous solutions and the relevant phenomenological binding constant of a number of ions [16]. However, the controversy regarding this issue remains, because other studies have shown that the binding of cations to PEO chains is absent in aqueous solutions [17].

Because of the importance of this issue, it is desirable to clarify the existence of cation binding to PEO chains in aqueous solutions. For this purpose, evidence of charging single PEO chains in aqueous solutions should be obtained at a single molecular level, such as the amount of effective charge and the single chain conformation. To fulfill this task, experimental investigations at the single molecular level should be effective. Based on the success of single-molecule fluorescence spectroscopy and microscopy in studying charged macromolecules, such as synthetic polyelectrolytes and biomacromolecules [18,19,20,21], an investigation into the binding of cations to PEO chains is conducted at a single molecular level, using fluorescence correlation spectroscopy (FCS) and photon-counting histogram (PCH). Experiments are performed in aqueous solutions and information is provided on the changes in chain conformation, the amount of effective charges, as well as the single-molecule electric potential. The results demonstrate the effect of cation binding to PEO chains in aqueous solutions, as well as the order of charging capacity of alkali cations.

## 2. Results and Discussion

**Figure 1:**
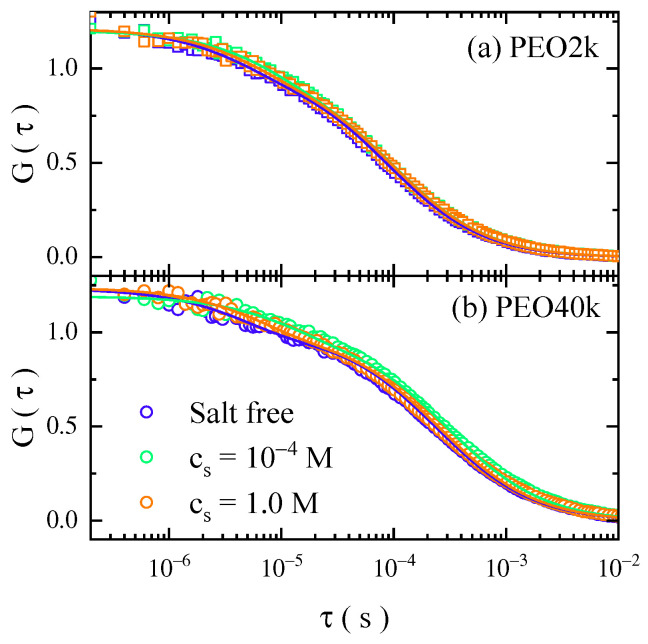
Auto-correlation functions of the fluorescence-labeled (**a**) PEO2k and (**b**) PEO40k in aqueous solutions under different salt concentrations: the salt-free condition, and salt concentrations of 1.0 × 10^−4^ and 1.0 M, respectively. The solid lines denote the result of the numerical fitting using a three-dimensional Brownian motion model. The fast dynamics around 10^−^^6^ s is due to the triplet state relaxation.

Figure 1 displays the typical autocorrelation functions, G(τ), of PEO2k and PEO40k labeled with a fluorescent molecule (Alexa 488) diffusing in aqueous solutions with different salt concentrations: the salt-free condition, and salt concentrations ($c_{s}$) of 1.0 × 10^−1^ and 1.0 M, respectively. (PEO2k and PEO40k were chosen as samples to check the possible effect of molecular weight. These two samples cover the range of more than one order of magnitude under the limit availability of the commercial provider, as detailed in the Materials and Methods Section). It was found that the data set shifted upon the introduction of external salt (NaCl), in general, the auto-correlation function shifted towards the long lag-time (τ) side when the $c_{s}$ was increased from the salt-free condition up to 1.0 × 10^−4^ M, and the further increase in the $c_{s}$ shifted the auto-correlation function back towards the short lag-time side. The shift of the data sets is small, and it is more obvious for the PEO40k case (Figure 1b) than PEO2k (Figure 1a). Such results demonstrate that the PEO single chain diffuses more slowly upon the addition of NaCl of a moderate concentration, compared to the case of the salt-free condition. The diffusion rate reaches its minimum value at $c_{s}$ of 1.0 × 10^−4^ M before it increases upon the further elevation of the salt level. The data sets are numerically fitted using a three-dimensional Brownian motion model, taking into account the fast dynamics due to the excited state, i.e., the triplet state effect (the details of the fitting are provided in the Materials and Methods Section).

**Figure 2:**
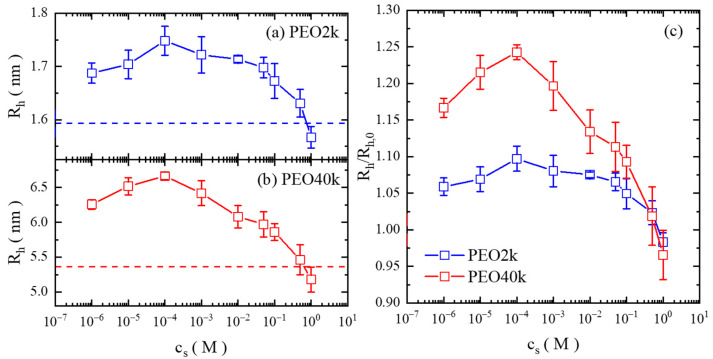
Values of the hydrodynamic radius ($R_{h}$) of the PEO single chain as a function of the salt concentration in aqueous solutions: (**a**) PEO2k and (**b**) PEO40k. The dashed lines denote the value under the salt-free condition of each case. (**c**) The normalized $R_{h}$ value (the ratio of $R_{h}$ over the value under the salt-free condition, $R_{h,0}$ ).

The changes of the diffusion rate and therefore the hydrodynamics radius ($R_{h}$) of the single PEO chain are summarized in Figure 2. It is immediately observed that the $R_{h}$ value increases from that under the salt-free condition when NaCl is introduced into the solution, and it reaches its maximum at $c_{s}$ of 1.0 × 10^−4^ M, after which it drops continuously. It is noted that at very high salt concentrations (1.0 M), the $R_{h}$ value becomes even smaller than that of the salt-free condition. Another important feature is the smaller difference of the relative change of $R_{h}$ of PEO2k than PEO40k; the increase in the $R_{h}$ value is ~25% at the salt concentration of 10^−4^ M, while that of PEO2k is ~10%.

Unlike the results obtained from the previous scattering experiments [14,15], the FCS data show the profound effect of added ions to the conformation of individual PEO chains in aqueous solutions—the chain expands from the salt-free condition upon the addition of salt of a moderate concentration, presumably due to the binding of the monovalent cation (Na^+^) to the PEO chain, so that the net charges are brought to the chain and the repulsion between the bound charges causes the chain to expand. With the further elevation of the salt level, the PEO chain shrinks, similar to the behavior of the polyelectrolyte, due to the effect of the promoted adsorption of chloride counter-ions and the screening effect [21,22].

The even smaller $R_{h}$ value at $c_{s}$ of 1.0 M than that of the salt-free condition indicates that the PEO chain is already charged without any salt addition. As demonstrated by previous studies [21,23], an elevated salt concentration results in enhanced counter-ion adsorption onto the charged main chain, weakening the repulsion along the chain and therefore making it contract. Provided that the PEO chain is not charged under salt-free conditions, its $R_{h}$ value should have been close to that in a high salt concentration, when the charges on the chain is neutralized to a significant extent. However, this is different to the current experimental observation.

**Figure 3:**
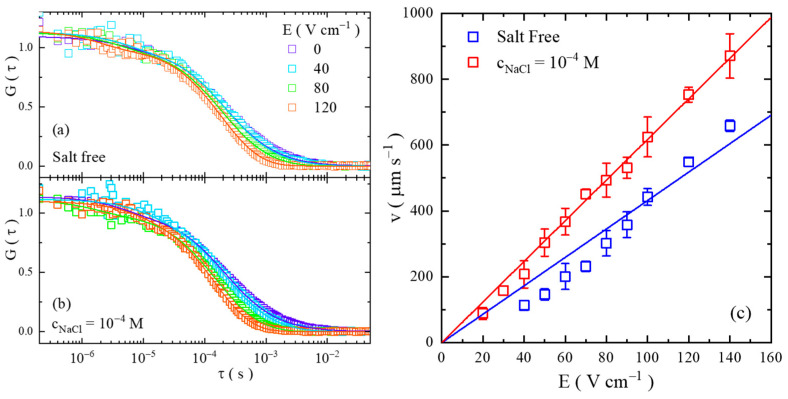
The auto-correlation function of PEO40k moving under the electric field. The values of the electric field strength are displayed accordingly in the figure. (**a**,**b**) are for the salt-free condition and salt concentration of 10^−4^ M, respectively. The solid lines denote the fitting results using a three-dimensional Brownian motion with directional motion. (**c**): the velocity of the PEO40k single chain (v) as a function of the strength of the electric field (E ). The solid lines denote the fitting of linearity.

The amount of the effective charge of the PEO chain is determined by examining the electrophoresis mobility of single PEO chains. The measurements were performed by FCS measurements with an electric field (e-field) applied to the samples. The details of the sample cell are provided in the Supplementary Materials. Figure 3a,b display a few typical data sets of the autocorrelation function of PEO40k in aqueous solutions under the e-field (Figure 3a for the salt-free condition and Figure 3b for the $c_{s}$ of 1.0 × 10^−4^ M). Compared to the situation without the e-field, the shape of the auto-correlation functions is changed by the application of the e-field; the longer lag-time portion is shifted towards the short lag-time side, while the portion of fast dynamics does not show notable changes, presenting the feature of the directional motion of the single PEO chains. Numerical fittings using the model of a three-dimensional Brownian motion with directional motion are performed, as denoted by the solid curves in Figure 3a,b, and the velocities of the directional motion are determined. The details of the fitting are provided in the Materials and Methods Section. The fitting results are summarized in Figure 3c, in which the migration velocity of the tPEO40k single chain scales linearly with the strength of he e-field (the directional motion under a low e-field is hard to be distinguished by FCS, and therefore only the data with notable directional motions are displayed. Additionally, as a result of this, the determination of velocity at a low e-field has greater uncertainties, and this makes the data deviate more from the linear fitting). In the current electrophoresis experiments, the electro–osmotic flow is ignored because of the large-length scale of the channel (the size of the rectangular cross-section is on the order of millimeters), and also because the channel surfaces were pre-adsorbed with excessive amounts of PEO to suppress the possible surface charges of the original materials (silica). Control experiments using sample cells with hydrophobically modified walls were conducted and similar results were obtained. The details are provided in the Supplementary Materials. In Figure 3c, the electrophoretic mobility ($\mu_{e}$) of the PEO chains under $c_{s}$ of 1.0 × 10^−4^ M is found to be higher than in the salt-free condition; its value is 6.2 × 10^−8^ m^2^⋅s⋅V^−1^ for a $c_{s}$ of 1.0 × 10^−4^ M and 4.3 × 10^−8^ m^2^⋅s⋅V^−1^ for the salt-free condition. These results indicate that a single chain of PEO40k carries more net charges under a $c_{s}$ of 1.0 × 10^−4^ M than the salt-free condition.

The number of effective charges on the PEO chains is estimated from the $\mu_{e}$ values. Due to the lower salt concentration used in these experiments, the electrophoretic mobility of the PEO chain can be expressed, at the large Debye length limit, by $\mu_{e}=q/6\pi \eta R_{h}$, where q is the net charge of the PEO chain and η is the viscosity of the solution [24,25,26]. The $R_{h}$ value of the PEO chain is assumed to be unchanged by the e-field. The number of net charges of the PEO40k chain is 24.2 (the charging extent of 2.7%) under the salt-free condition and 43.5 (the charging extent of 4.7%) in the salt concentration of 1.0 × 10^−4^ M.

**Figure 4:**
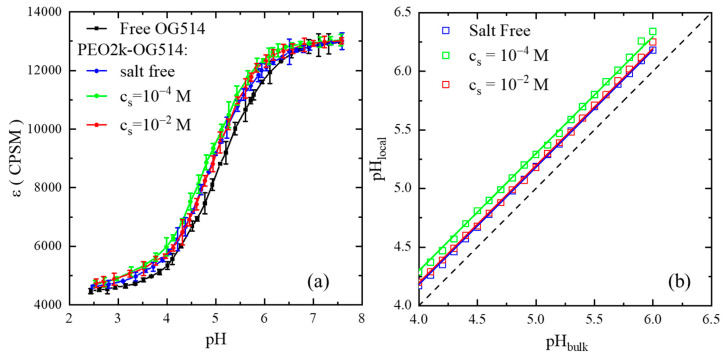
(**a**) Values of the molecular brightness (ε) of OG514 as a function of pH in the solution when it is free and attached to the chain-end of PEO2k under different salt concentrations. (**b**) Values of local pH at the PEO chain-end as a function of the pH value of the solution. The dashed line denotes the results of free OG514.

PCH experiments were conducted on samples labeled with the pH-responsive OG514 fluorophore to 31. (The adjustment of the pH value was conducted through the addition of HCl and NaOH, instead of a buffer solution, in order to suppress the possible additional effect of external salt. Additionally, the addition of NaCl in the concentration range under investigation does not change the molecular brightness of free OG514, as detailed in the Supplementary Materials). The data set of ε is further shifted upon the introduction of NaCl at the concentration of 1.0 × 10^−4^ M (the green data set), beyond which the data set is shifted backwards at the salt concentration of 1.0 × 10^−2^ M (the red data set) and becomes indistinguishable from that of the salt-free condition. By comparing the ε data of OG514-PEO2k with those of free OG514 (the master curve), the local pH value at the PEO chain-end is determined and the data are summarized in Figure 4b. It is immediately noticed that, under the salt-free condition, the local pH values at the PEO chain’s vicinity are higher than those in the bulk solution, indicating a lower concentration of hydronium ions near the PEO chain. This result demonstrates that the PEO chain is positively charged in the aqueous solution, as the hydronium ions are slightly depleted at the chain vicinity due to electrostatic repulsion.

The electric potential of the PEO chain is calculated based on the fitting by applying the universal Boltzmann distribution [20,27,28]: ${\left[H^{+}\right]}_{local}={\left[H^{+}\right]}_{bulk}exp\left(-\frac{e\psi }{k_{B}T}\right)$, where ${\left[H^{+}\right]}_{local}$ and ${\left[H^{+}\right]}_{bulk}$ are the concentrations of hydronium ions at the vicinity of the PEO chain and that in the bulk solution, respectively; e is the element charge; ψ is the electric potential; $k_{B}$ is the Boltzmann constant; and T is the absolute temperature. The alternative expression is $pH_{local}=pH_{bulk}+0.43\frac{e\psi }{k_{B}T}$, where $pH_{local}$ and $pH_{bulk}$ are the local pH values at the PEO chain’s vicinity and that of the bulk solution, respectively. ($pH_{local}$ and $pH_{bulk}$ are defined as $-log{\left[H^{+}\right]}_{local}$ and $-log{\left[H^{+}\right]}_{bulk}$, respectively). The fitting results are demonstrated in Figure 4b and the values of the electric potential of the PEO single chain are 10.8 mV for the salt-free condition, 17.8 mV for the $c_{s}$ of 1.0 × 10^−4^ M, and 11.8 mV for $c_{s}$ of 1.0 × 10^−2^ M. These values show that the PEO chain is charged in aqueous solutions, even in the salt-free condition, and it should be the result of the binding of hydronium ions. The elevation of ionic strength by adding external salt makes it more charged at intermediate salt concentrations, due to the binding of sodium ions, whose concentration overwhelms that of the hydronium ions. A further increase in the salt concentration reduces the net charge, due to the neutralization effect of the counter-ions, i.e., the chloride ions [21]. It is interesting to compare these values with previously reported values of fully charged polyelectrolytes with a similar degree of polymerization, such as sodium polystyrene sulfonate, whose absolute single-chain electric potential is about ten times greater [20,27]. This indicates that PEO is much less charged in aqueous solutions than polyelectrolytes, consistent with the result of single-chain electrophoresis experiments. (The investigations into the molecular weight dependence of electric potential were not successful because the molecular brightness of OG514 attached to longer PEO chains was affected, possibly due to the change of the local dielectric constant when the fluorophore started to have a probability of residing inside the coiled chains. Details are provided in the Supplementary Materials).

Further investigations into the effect of cation identity were conducted by electrophoresis, choosing alkali chloride salts, such as LiCl, NaCl, KCl, and CsCl. The results of electrophoresis are summarized in the Supplementary Materials (Figure S4). The electrophoretic mobility of PEO40k in LiCl, NaCl, KCl, and CsCl solutions at the $c_{s}$ of 1.0 × 10^−4^ M are 5.5 × 10^−8^, 6.2 × 10^−8^, 7.8 × 10^−8^ and 6.5 × 10^−8^ m^2^⋅s⋅V^−1^, respectively. By measuring the $R_{h}$ of PEO40k in all of these solutions, the amounts of net charges of PEO40k are 37.5, 43.5, 55.4 and 45.8 in LiCl, NaCl, KCl and CsCl, respectively (detailed in the Supplementary Materials, Table S1), demonstrating the capacity order of charging the PEO chain as Li^+^ < Na^+^ < Cs^+^ < K^+^. This order follows the general feature of the Hofmeister series ions, with the exception of the Cs^+^ ion [29,30]. Therefore, the results show that the binding strength of specific ions may vary, depending on the counterpart molecules.

## 3. Conclusions

PEO chains are found to be charged in aqueous solutions, due to the binding of cations, such as hydronium and alkali ions. The binding of hydronium ions charges PEO chains, even in the salt-free condition. The addition of NaCl salt in intermediate concentrations, further charges the chain due to the binding of Na^+^ ions as a result of their overwhelmingly higher concentration, compared to that of H^+^ ions. At a high enough salt concentration, the effective charge of the PEO chain is reduced, due to the neutralization effect by the enhanced adsorption of the Cl^−^ counter-ions. The charging effect makes the PEO chains expand, as demonstrated by the increase in the hydrodynamic radius upon the addition of NaCl salt, measured at the single molecular level by FCS. PCH measurements demonstrate that the PEO chains are positively charged, as a result of cation binding. The single molecule electrophoresis experiment by FCS exposes the amount of effective charge of the PEO, showing that the PEO chains are weakly charged. The effect of cation identity on the charging of the PEO chain is discovered, following the order of Li^+^ < Na^+^ < Cs^+^ < K^+^.

## 4. Materials and Methods

### 4.1. Materials

PEO (M_w_ = 2000 g·mol^−1^, M_w_/M_n_ = 1.04, M_w_ = 10,000 g·mol^−1^, M_w_/M_n_ = 1.05 and M_w_ = 40,000 g·mol^−1^, M_w_/M_n_ = 1.07) terminated with an amino group at one of its chain-ends was purchased from Polymer Source, Montreal, Canada. The PEO samples were fluorescence-labeled by chemically attaching a fluorescent molecule to the chain end. For different purposes, two types of fluorescent molecules were chosen—Alexa Fluor 488 succinimidyl ester (Molecular Probes, Waltham, MA, USA) and Oregon Green 514 succinimidyl ester (Invitrogen, Waltham, MA, USA). The former (Alexa488) is not pH responsive and was used for measuring the hydrodynamic radius of PEO single chains by FCS. The latter (OG514) is pH responsive and was adopted to measure the local concentration of hydronium around PEO chains by PCH, providing information for the polarity of the charges of the PEO chain [18,20,21,22,27]. After chemical reactions for labeling, the samples were purified by gel chromatography with a polyacrylamide filler and multiple rounds of ultrafiltration using a filter with the cut-off molecular weight of 2000 Dalton. The complete removal of unreacted fluorescent molecules was proven by the absence of a detectable fluorescence signal from the residual solution measured by single-photon counting methods. It is noted that after the labeling process, the amino group at the PEO chain-end reacted with the probe molecule, forming an amide bond connecting the PEO chain and the probe. Therefore, the original amino group should not have an effect in the experiment. The concentrations of PEO2k and PEO40k were kept as ~5.0 × 10^−9^ M.

### 4.2. Fluorescence Correlation Spectroscopy (FCS)

Considering that the principle of FCS has been reviewed multiple times [31,32,33,34,35], a brief description is provided here. An FCS setup is based on an optical microscope with a confocal configuration. The fluorescence signal inside the confocal volume was detected by single-photon counting techniques. Because of the diffusive motion of the molecules under investigation, the fluctuation of the fluorescence signal from the confocal volume was generated as the fluorescent molecule moved in and out of the confocal volume. The autocorrelation function of the fluorescence fluctuation is defined as $G\left(\tau \right)=\delta I\langle (t)\delta I(t+\tau )\rangle /{\langle I(t)\rangle }^{2}$, where I(t) denotes the temporal profile of fluorescence intensity and τ is the time lag. Such an autocorrelation function can be numerically fitted, based on the Gaussian profile of the laser intensity distribution and the three-dimensional Brownian motion model, expressed as $G\left(\tau \right)=N^{-1}{\left(1+4D\tau /w_{0}^{2}\right)}^{-1}{\left(1+4D\tau /z_{0}^{2}\right)}^{-1/2}$, where $w_{0}$ is the radius of the confocal volume perpendicular to the optical axis, $z_{0}$ is the half-length in parallel direction, and D is the translational diffusion coefficient. The values of $w_{0}$ and $z_{0}$ are calibrated by measuring the diffusion of standard samples, such as the diluted aqueous solution of Rhodamine 6G, whose D value is 414 μm^2^ s^−^^1^ at 25 °C. With the correct calibration of the confocal volume, the D value of the unknown samples can be measured. With D measured, the hydrodynamic radius $R_{h}$ of the diffusing molecule is determined by applying the Stokes–Einstein equation, $R_{h}=k_{B}T/6\pi \eta D$, where $k_{B}$, T and η are the Boltzmann constant, the absolute temperature, and the viscosity of the medium, respectively. In the current study, the aqueous solution is the medium and its viscosity can be estimated by the relation of $\eta /\eta_{0}=1+A\sqrt{c}+Bc$, where η and $\eta_{0}$ are the water viscosity with and without the addition of salt, respectively, and the values of A and B are 0.0062 and 0.0793, respectively. [36,37]

In addition to being capable of measuring diffusion inside solutions under thermo-equilibrium, FCS can also measure the velocity of directional motions of single molecules [24,38,39], and this function is capable of measuring the speed of the charged molecules making directional motions under the electric field in the current study. The standard autocorrection function of molecules making both diffusive and directional motions can be described as $G\left(\tau \right)=N^{-1}{\left(1+4D\tau /w_{0}^{2}\right)}^{-1}{\left(1+4D\tau /z_{0}^{2}\right)}^{-1/2}exp\left(-{\left(\tau /\tau_{f}\right)}^{2}{\left(1+\tau /\tau_{f}\right)}^{-1}\right)$, and the migration velocity of the molecules is $v=w_{0}/\tau_{f}$, where $\tau_{f}$ is the translocation time of the molecule across the confocal volume.

The FCS setup used in the current study is a commercial one (LSM780, Carl Zeiss, Köln, Germany) and a water-immersion objective lens (40×Plan-Neofluar, numerical aperture = 1.20) was used.

### 4.3. Photon-Counting Histogram (PCH)

PCH measures the fluorescence photon counts emitted by the diffusing molecules inside the confocal volume, using the same setup as FCS [40,41]. In brief, PCH analyzes the distribution of fluorescence photon counts, i.e., the probability to detect k-photon counts, p(k), per sampling time in the observation time $p\left(k,t,T\right)=\int_{0}^{\infty }\left({\left(\eta_{w}W\left(t\right)\right)}^{k}e^{-\eta_{w}W\left(t\right)}/k!\right)p\left(W\left(t\right)\right)dW\left(t\right)$, where p(k,t,T) is the probability of observing k-photoelectron events at time t, $W\left(t\right)=\int_{t}^{t+T}\int I\left(r,t\right)dAdt$ is the light energy reaching the detector, $\eta_{w}$ is the efficiency of the detector, and T is the integration time. When the fluorescence source is stationary, the photon-count histogram exhibits the standard Poisson distribution. In the case of diffusing fluorescent molecules, the random motion creates an inhomogeneous excitation profile, causing the additional broadening of the photon-counting histogram. The probability of observing k-photoelectron events at the three-dimensional Gaussian excitation-detection volume $V_{0}$ can be expressed by $\int_{V_{0}}Poi\left(k,\varepsilon PSF\left(r\right)\right)dr=\frac{1}{V_{0}}\frac{\pi w_{0}^{2}z_{0}}{k!}\int_{0}^{\infty }\gamma \left(k,\varepsilon e^{-2x^{2}}\right)dx$, where ε is the brightness of the fluorescent molecule. By fitting the data with a “super-Poisson” distribution function, the number of molecules within the observation volume and the molecular brightness in units of detected photon-counts per second per molecule (CPSM) are obtained [40,41].

The PCH measurements in the current study were performed on a home-built set-up based on an inverted optical microscope (IX-71, Olympus, Japan) [18,20,21,22,27,35]. A water-immersion objective lens (UPlanApo 60×, numerical aperture = 1.20) was used. In all of the experiments, the intensity of the excitation light at the sample stage was in the order of μW, in order to obtain a high enough signal-to-noise ratio without creating a photobleaching effect.

## Data Availability

The data presented in this study are available on request from the corresponding author. The data are not publicly available due to limited space.

## Supplementary Materials

The following Supplementary Materials can be downloaded at: https://www.mdpi.com/article/10.3390/gels8040213/s1, Figure S1: The illustration of the sample cell; Figure S2: Values of molecular brightness of free OG514 as a function of salt concentration under different pH values; Figure S3: The molecular brightness of OG514 attached at the chain-end of PEO40k (OG514-PEO40k) as a function of pH value in the solution; Figure S4: The velocity of the PEO40k single chain as a function of the strength of the e-field in different salt solutions; Figure S5: Correlation function of the PEO40k sample under the e-field and salt-free condition inside the sample cell with hydrophobic walls; and Figure S6: Temperature of the sample as a function of the e-field strength. Table S1: Hydrodynamic radius, electrophoretic mobility and effective charge of the PEO40k sample in aqueous solution with different alkali chloride salts.
