# Supplementary material for: Poly(ethylene oxide) Is Positively Charged in Aqueous Solutions
Source: Gels. 2022 Mar 31;8(4):213. doi: 10.3390/gels8040213 (PMC9029200; doi:10.3390/gels8040213)
Supplement: Supplementary file 1 [file gels-08-00213-s001.zip › gels-1628784-supplementary.pdf]

gels

Article

Poly(ethylene oxide) is Positively Charged in Aqueous Solutions

Chao Zhou [1,2], Chunda Ji [1,2], Yuchen Nie [1,2], Jingfa Yang [1,2] and Jiang Zhao [1,2,*]

[1] Beijing National Research Center for Molecular Sciences, Institute of Chemistry, Chinese Academy of Sciences, Beijing 100190, China; zhouchao@iccas.ac.cn (C.Z.); jichunda@iccas.ac.cn (C.J.); nieyuchen19@mails.ucas.ac.cn (Y.N.); yangjf@iccas.ac.cn (J.Y.)
[2] University of Chinese Academy of Sciences, Beijing 100049, China
* Correspondence: jzhao@iccas.ac.cn

Supplementary Materials

1. Sample Cell

The structure of the sample cell for electrophoresis measurements by FCS is illustrated in Figure S1. The objective lens of the microscope is facing the sample cell from the bottom.

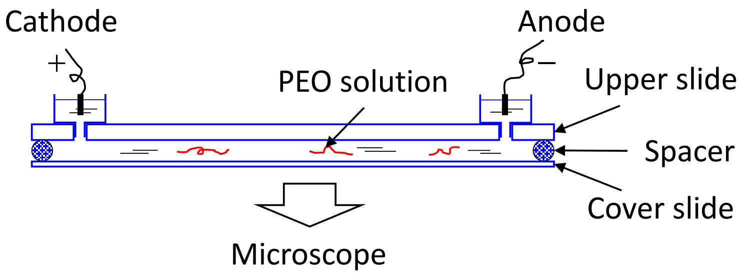

Figure S1. The illustration of the structure of the sample cell for electrophoresis measurement by FCS.

2. Proof of Unchanged Molecular Brightness of Free OG514 within the Salt Concentration Under Investigation

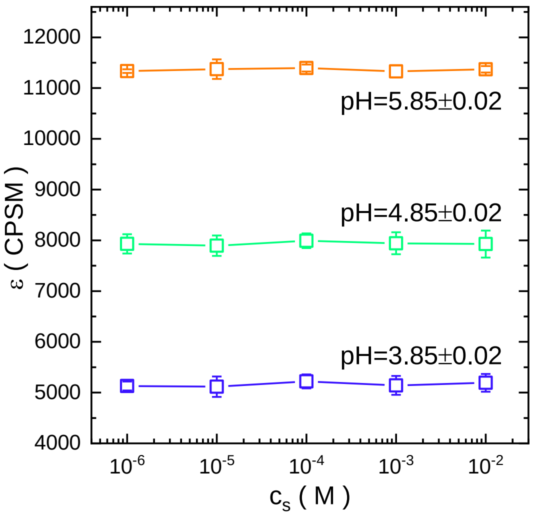

Figure S2. Values of molecular brightness of free OG514 as a function of salt concentration under different pH values. The salt concentration was adjusted to be within the range of the current investigation.

Concentration under Investigation

Control experiments were performed to check whether the molecular brightness experiences a change when the salt concentration of the solution is varied. The data are provided in Figure S2, showing that the molecular brightness does not show noticeable variation within the concentration range of the current investigation.

3. Molecular Brightness of OG514 Labeled at PEO of Different Molecular Weight

The molecular brightness of OG514 (ε) attached to PEO chain end is found to be dependent on the molecular weight of PEO. Figure S3a displays the ε values of OG514 attached to PEO40k as a function of pH value of the solution. As a comparison, the data set of free OG514 is also displayed. The data show that OG514-PEO40k exhibits a much smaller pH response than free OG514. Although at low pH region, the ε values are higher when the probe is attached to PEO40k, it deviates from the pH-responsiveness curve and ends up with a lower ε value at high pH region, compared with the case of free OG514. The ε values under pH 9.35 of free OG514 and OG514 attached to PEO2k, PEO10k and PEO40k are summarized in Figure S3b, in which the ε value decreases with the increase of the molecular weight of PEO. Such a suppression of the pH responsiveness of OG514 is presumably attributed to the decrease of local dielectric constant within the random coil of PEO. Therefore, the PCH experiment was conducted with PEO2k sample only, which does not show a notable effect to its pH responsiveness.

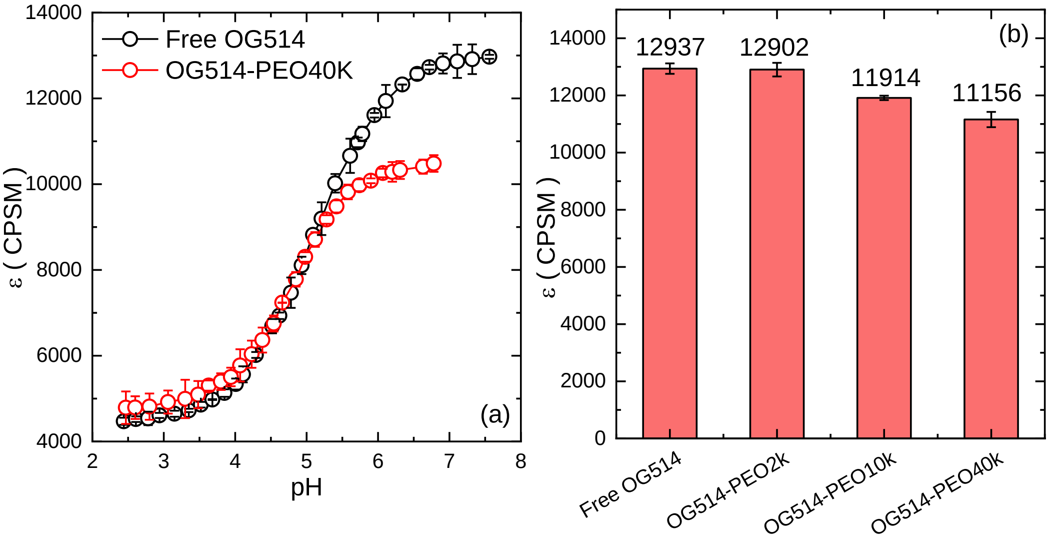

Figure S3. a) The molecular brightness of OG514 attached at the chain end of PEO40k (OG514-PEO40k) as a function of pH value in the solution. The data set of free OG514 is displayed for a comparison. (**b**) The molecular brightness of OG514 at pH of 9.35 when it is free and attached to PEO2k, PEO10k, PEO40k, respectively.

4. The Effect of Ion Identity

The effect of cation identity is investigated by electrophoresis using alkali chloride salts—LiCl, NaCl, KCl and CsCl, with the concentration of 1.0 × 10^{-4} M. The results are summarized in Figure S4, in which the migration velocity of PEO scales linearly with the E-field strength, yielding the values of electrophoretic mobility of 5.5 × 10^{-8}, 6.2 × 10^{-8}, 7.8 × 10^{-8} and 6.5 × 10^{-8} $m^2 \cdot s \cdot V^{-1}$ for LiCl, NaCl, KCl and CsCl solutions, respectively.

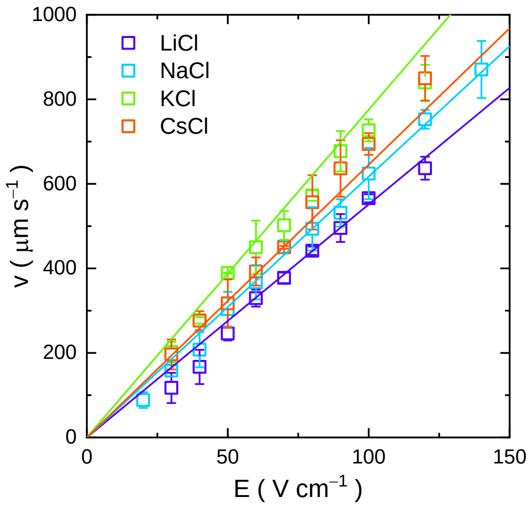

Figure S4. The velocity of PEO40k single chain (v) as a function of the strength of electric field (E) in different salt solutions with the concentration of 1.0 × 10^{-4} M. The types of the salt adopted are specified in the figure. The solid lines denote the fitting of linearity.

The R_h values of PEO40k in all solutions are measured, with which the amount of net charges of PEO40k in all solutions are determined, as summarized in Table S1.

Table S1. Hydrodynamic radius, electrophoretic mobility and effective charge of PEO40k sample in aqueous solution with different alkali chloride salts.

Ion Identity	Li^+	Na^+	K^+	Cs^+
R_h (nm)	6.5	6.7	6.8	6.7
μ_e (10^{-8}·m^2·s·V^{-1})	5.5	6.2	7.8	6.5
q	37.5	43.5	55.4	45.8

5. The Results in Sample Cell with Hydrophobic Walls

FCS experiments under E-field on PEO40k sample was inside sample cell with its wall modified to be hydrophobic. The purpose is to suppress possible electro-osmotic flow. The wall of the sample cell is modified by growing a hydrophobic monolayer. For surface treatment, the sample cell was ultrasonically cleaned with acetone, ethanol, and de-ionized water for 10 min, respectively. Afterwards, the cell was treated in oxygen plasma for 15 min to remove residual organic substances and carbon on the surface. The treated sample cell was then immersed in octadecyltrichlorosilane (OTS) cyclohexane solution with a concentration of 0.1% for 12 h. After this process, the sample cell was ultrasonically cleaned with cyclohexane, acetone and de-ionized water for 10 min successively, followed by purging with high-purity nitrogen. The hydrophobicity of the sample cells was characterized by the water contact angle measurement on quartz coverslips treated in parallel. The water contract angle is 118.8 °.

The correlation functions of PEO40k sample under different E-field and salt-free condition are displayed in Figure S5a and the E-field strength dependence of the velocity is shown in Figure S5b. The corresponding mobility is measured to be 4.2 × 10^{-8} m^2·s·V^{-1}. This value is close to the value measured in the sample cell with walls pre-adsorbed with PEO.

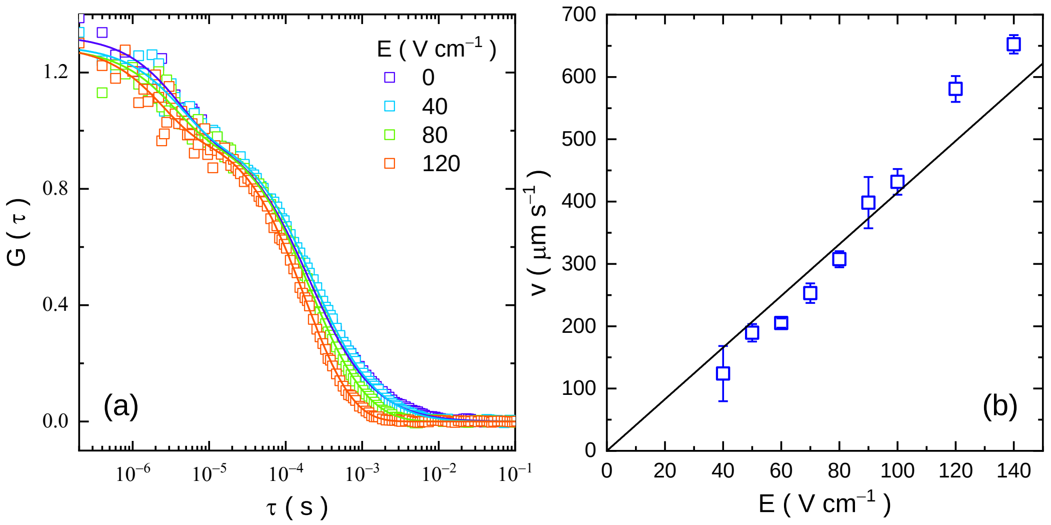

Figure S5. Correlation function of PEO40k sample under E-field and salt-free condition inside the sample cell with hydrophobic walls.

6. *Temperature of the Sample when E-field is Applied*

The temperature of the sample cell is monitored and the results are displayed in Figure S6, in which a slight increase of temperature is observed–under most of the E-field strength, the increase of temperature is within 1 °C and under the maximum E-field (140 V cm^{-1}), the increase is 1.3 °C.

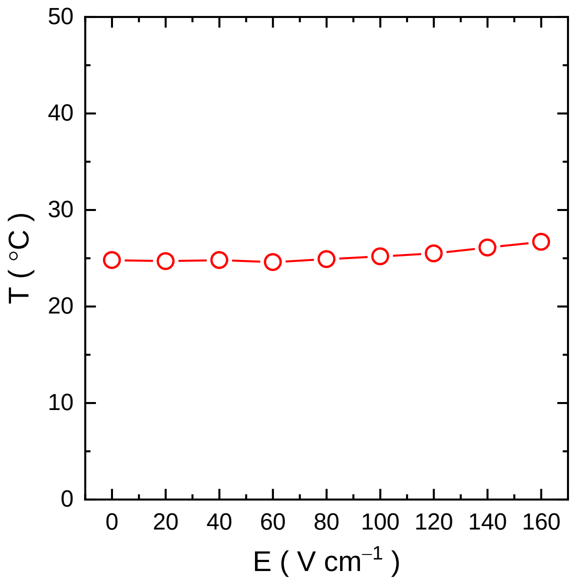

Figure S6. Temperature of the sample as a function of the E-field strength.
